# The N- and C-terminal carbohydrate recognition domains of *Haemonchus contortus* galectin bind to distinct receptors of goat PBMC and contribute differently to its immunomodulatory functions in host-parasite interactions

**DOI:** 10.1186/s13071-017-2353-8

**Published:** 2017-09-05

**Authors:** MingMin Lu, XiaoWei Tian, XinChao Yang, Cheng Yuan, Muhammad Ehsan, XinChao Liu, RuoFeng Yan, LiXin Xu, XiaoKai Song, XiangRui Li

**Affiliations:** 1 College of Veterinary Medicine, Nanjing Agricultural University, Nanjing, Jiangsu People’s Republic of China; 2 College of Veterinary Medicine, Jiangsu Agri-animal Husbandry Vocational College, Taizhou, Jiangsu People’s Republic of China

**Keywords:** Galectin, *Haemonchus contortus*, Carbohydrate recognition domain, PBMC, Receptor, Immunomodulatory

## Abstract

**Background:**

Hco-gal-m is a tandem-repeat galectin isolated from the adult worm of *Haemonchus contortus*. A growing body of studies have demonstrated that Hco-gal-m could exert its immunomodulatory effects on host peripheral blood mononuclear cells (PBMC) to facilitate the immune evasion. Our previous work revealed that C-terminal and N-terminal carbohydrate recognition domains (CRD) of Hco-gal-m had different sugar binding abilities. However, whether different domains of Hco-gal-m account differently for its multiple immunomodulatory functions in the host-parasite interaction remains to be elucidated.

**Results:**

We found that the N-terminal CRD of Hco-gal-m (MNh) and the C-terminal CRD (MCh) could bind to goat peripheral blood mononuclear cells by distinct receptors: transmembrane protein 63A (TMEM63A) was a binding receptor of MNh, while transmembrane protein 147 (TMEM147) was a binding receptor of MCh. In addition, MCh was much more potent than MNh in inhibiting cell proliferation and inducing apoptosis, while MNh was much more effective in inhibiting NO production. Moreover, MNh could suppress the transcription of interferon-γ (IFN-γ), but MCh not.

**Conclusions:**

Our data suggested that these two CRDs of Hco-gal-m bind to distinct receptors and contributed differently to its ability to downregulate host immune response. These results will improve our understanding of galectins from parasitic nematodes contributing to the mechanism of parasitic immune evasion and continue to illustrate the diverse range of biological activities attributable to the galectin family.

**Electronic supplementary material:**

The online version of this article (10.1186/s13071-017-2353-8) contains supplementary material, which is available to authorized users.

## Background

The gastrointestinal nematode *Haemonchus contortus*, a voracious blood-sucker residing in the abomasum, is one of the most economically important parasites for small ruminants [1]. Infection with *H. contortus* can lead to severe anemia, diarrhea, dehydration and even death of the hosts and, therefore, causes major economic losses in the livestock industry worldwide [2]. Galectins are an ancient family of glycan-binding proteins (GBPs) characterized by a conserved carbohydrate recognition domain (CRD) sequence in common, which can function intracellularly and be secreted to bind to the ligands of cell surface and pathogens [3]. Among GBP-ligand interactions that engage a diverse set of biological processes, galectins perhaps regulate the widest range of cellular functions, such as cell proliferation, activation, apoptosis and cytokine secretion, which made galectins key regulators of the immune response [4–6].

Galectins are expressed by many cell types, and parasites can also produce galectins or galectin-like proteins [7]. Given the wide distribution in both parasites and host, galectins play a central role in host-parasite interactions [8]. A recent report indicated that human galectin-1 could prevent *Trypanosoma cruzi* induced apoptosis of in HL-1 cardiac cells and reduce its infectivity [9]. In addition, mouse galectin-3 could upregulate ROS generation by neutrophils to control parasite growth during early *Toxoplasma gondii* infection [10]. In contrast, parasites may evolve to evade the inhibitory effect of galectins or directly produce the galectins to ensure their invasion and survival. It was suggested that *Trichomonas vaginalis* was capable of altering human galectin-1 and -3 bioavailability, via the binding of lipophosphoglycan to ceramide phosphoinositol glycan core to modulate epithelial immunity [11]. Notably, galectin from *Dirofilaria immitis* could bind plasminogen and enhance plasmin generation to activate the fibrinolytic system, as a survival mechanism to avoid the formation of blood clots in its nearby environment [12].

In previous research, we reported Hco-gal-f (GenBank AY253331) and Hco-gal-m (AY253330), two isoforms of galectins derived from female (f) and male (m) *H. contortus* [13]. They can induce same biological effects, including suppressing the hemagglutination of goat erythrocytes [14], inducing cell apoptosis and altering cytokine mRNA transcription [15, 16]. Meanwhile, proteomic and transcriptional analyses indicated that rHco-gal-m/f could inhibit the activations of free radical producing pathway, NFκB pathway, ubiquitin-proteasome pathway, VEGF pathway in PBMCs in vitro [17]. Our research further revealed that transmembrane protein 147 (TMEM147) and transmembrane protein 63A (TMEM63A) were identified to be receptors of Hco-gal-m/f by yeast two-hybrid (YTH) screening. Furthermore, knockdown of the tmem63a and tmem147 gene by RNA interference (RNAi) revealed that the interaction of Hco-gal-m/f with TMEM63A and the interaction of Hco-gal-m/f with TMEM147 mediated similar effects on PBMC, including cell proliferation, phagocytosis, nitric oxide production, transcription of transforming growth factor-β1 (TGF-β1) and interleukin-10 (IL-10) [18, 19]. All these findings suggested that Hco-gal-m/f contributed to the regulation of host immune response or parasite immune evasion.

Hco-gal-m/f belongs to the tandem-repeat (TR) galectin subfamily with two CRDs in the N- and C-terminal regions and shows 20–34% sequence identity with other subfamily members (galectin-4, -6, -8, -9, -12) of humans and other mammals. Recent studies demonstrated that the individual CRDs of tandem repeat galectins may retain different biological activities. From the functional standpoint, the most striking example is that C-terminal domain of human Gal-4 and -8 could kill blood group B positive *Escherichia coli* (BG B^+^ *E. coli*) through the recognition of blood group antigens, while the N-terminal domain of Gal-4 could only recognize BG B^+^ *E. coli* but not affect its viability, and the N-terminal domain of Gal-8 could not even recognize blood group antigens [20]. Additional studies suggested that the C-terminal CRD of human galectin-9, but not N-terminal CRD, was the dominant factor of receptor recognition and death pathway signaling [21], while the N-terminal CRD was much more potent in the activation of dendritic cells by inducing high levels of p38 and AKT phosphorylation [22]. However, there is a paucity of published information regarding the key differences for the multiple CRDs of tandem-repeat parasite galectins.

In our previous research, we found that the C-terminal CRD of Hco-gal-m/f had higher sugar binding ability than the N-terminal CRD [23]. However, it is still unclear whether different domains of Hco-gal-m/f account differently for its immune suppressive functions to facilitate the immune evasion. Here, we found that the N-terminal CRD of Hco-gal-m (MNh) identified TMEM63A, while the C-terminal CRD (MCh) preferred TMEM147. In addition, we directly compared MNh, MCh, and the full-length Hco-gal-m induced host immune response with regard to cell proliferation, cell apoptosis, nitric oxide production and cytokine transcription and found that MNh and MCh contributed differently to these functions. The results presented herein will further elucidate the mechanism underlying the immune evasion and modulation induced by parasitic galectins and improve our understanding of the complex biological roles of tandem-repeat galectin subfamily.

## Methods

### Animals

Local crossbred goats (3–6 month-old), fed with hay and whole shelled corn, from the teaching and research flock at Nanjing Agricultural University were housed indoor in pens and provided with water *ad libitum*. All goats were dewormed twice at 2 week intervals with levamisole (8 mg/kg of bodyweight), given orally at the time of housing, to remove naturally acquired strongylid infections. Following standard parasitological techniques, a fecal sample from each goat was examined by microscopy for helminth eggs after 2 weeks. Goats manifesting no eggs were used in the subsequent study and daily health observations were performed throughout the experiment. The isolation and culture of goat PBMC were performed as previously described [18]. Three biological replicates (three goats), each consisting of three technical replicates (three replicates for each goat), were run for immune and functional studies including immunofluorescence assays, co-immunoprecipitation assays, cell proliferation, nitric oxide production, apoptosis and transcriptional analysis.

Sprague Dawley (SD) rats (body weight ~150 g) were purchased from the Experimental Animal Center of Jiangsu, PR China (Qualified Certificate: SCXK 2008–0004) and were raised in a sterilized room and fed sterilized food and water.

### Preparation of recombinant proteins

The recombinant proteins were expressed and purified as previously described [24]. In brief, the PCR products of two CRDs of Hco-gal-m were cloned into the pET-32a prokaryotic expression vector (Additional file 1: Table S1). *Escherichia coli* BL21 cells containing the constructed plasmids were cultured in Luria-Bertini medium with ampicillin (100 μg/ml) and induced with Isopropyl-β-D-thiogalactopyranoside (IPTG) at 37 °C for 5 h to express the recombinant proteins. The histidine-tagged fusion protein was purified from the supernatant of bacterial lysates using the HisBind® Resin Chromatography kit (Merck, Darmstadt, Germany). The purity of the protein preparation was determined by SDS-PAGE (Additional file 2: Figure S1) and protein concentrations were determined by Bradford method. Lipopolysaccharide (LPS) was depleted from the recombinant proteins using Detoxi-Gel Affinity Pak prepacked columns (Pierce, Rockford, USA). The purified proteins were stored at -70 °C until to be used. The *E. coli* containing empty plasmid were cultured and the cell lysates were purified under the same conditions.

### Generation of polyclonal antibody

To generate polyclonal antibodies against rMNh or rMCh, 0.3 mg of purified proteins mixed with Freund’s complete adjuvant (1:1) were injected subcutaneously into SD rats. After the first injection, SD rats were then boosted four times with the same dose at 2-week intervals. One week after the last injection, the serum containing specific anti-MNh/MCh antibodies was collected and then stored at -70 °C for later use. Rat anti-TMEM147-O IgG and rat anti-TMEM63A-NO IgG were from Yan Li and Cheng Yuan, respectively [18, 19].

### Immunofluorescence assay

Confirmation of interaction was performed by an immunofluorescence assay (IFA) as previously described [25]. Briefly, freshly isolated PBMC were incubated with empty recombinant pET-32a protein, rMNh and rMCh, respectively, for 1 h at 37 °C. To minimize background staining, washed cells fixed with 4% paraformaldehyde were treated with blocking solution (4% BSA in PBS) for 30 min. Then cells were incubated with negative rat IgG (control) or rat anti-pET-32a protein/MNh/MCh polyclonal antibody (1:100 dilution) for 2 h, followed by staining with the secondary antibody (1:100 dilution) coupled to the fluorescent dye Cy3 (Beyotime, China) for 1 h. 2-(4-amidinophenyl)-6-indole carbamidinedihydrochloride (DAPI, 1.5 μM; Sigma, MO, USA) were used for nuclear staining. Ultimately, the binding was determined by checking the staining patterns with a 100× oil objective lens on a laser scanning confocal microscope (LSM710, Zeiss, Jena, Germany) and digital images were captured using the Zeiss microscope software package ZEN 2012 (Zeiss, Jena, Germany).

### Split ubiquitin protein-protein interaction analysis

Split-ubiquitin YTH assays were used to identify interaction between the two CRDs to TMEM63A or TMEM147, following the protocol of DUAL membrane pairwise interaction kit (Dualsystems Biotech, Schlieren, Switzerland). Full-length cDNAs of TMEM63A and TMEM147 were cloned in frame into the Cub domain bait vector pBT3-STE and pBT3-SUC, respectively (Additional file 1: Table S2). The coding regions of MNh and MCh were cloned in frame in the Nub domain prey vector pPR3-N (Additional file 1: Table S2). Different pairs of bait and prey vectors were co-transformed into yeast reporter strain NMY51. Transformed colonies were incubated for growth of positive transformants on SD-LW selective medium. Several independent positive transformants were re-cultured in SD-LW liquid medium at 30 °C until the OD546 of the cultures reached 1.0. For protein-protein interaction assays, 5 μl of each diluted cultures (1:10, 1:100 and 1:1000) were applied on SD-LW and SD-LHAW selection plates, respectively, and incubated at 30 °C for 2–3 days. Three independent experiments, each consisting of three replicates, were carried out.

### Co-immunoprecipitation (co-IP) assays

To validate protein-protein interactions, co-IP assays were performed as previously described [18]. The goat PBMC incubated with rMNh or rMCh for 12 h were washed, pelleted and lysed. After pretreatment, triplicate 1 mg cell lysates for IP were incubated overnight at 4 °C with the following: rat anti-TMEM63A-NO IgG for input samples, rat anti-MNh IgG for IP samples, and normal rat IgG (Santa Cruz Biotechnology, Dallas, Texas, USA) for negative control samples in forward IP; rat anti-TMEM147-O IgG for input samples, rat anti-MCh IgG for IP samples, and normal rat IgG for negative control samples also in forward IP; rat anti-MNh IgG for input samples, rat anti-TMEM63A-NO IgG for IP samples and normal rat IgG for negative control samples in reverse IP; rat anti-MCh IgG for input samples, rat anti-TMEM147-O IgG for IP samples and normal rat IgG for negative control samples also in reverse IP. Immune complexes were precipitated using 20 μl Protein A/G PLUS-Agarose (Santa Cruz Biotechnology, Texas, USA).

After four rounds of washing, the pellets were re-suspended in 1× SDS-PAGE loading buffer. The resulting protein samples were separated by 12% SDS-PAGE gel and electro-transferred onto nitrocellulose membranes. Membranes were probed with rat anti-TMEM147-O/TMEM63A-NO IgG for forward IP experiments and rat anti-MCh/ MNh IgG for reverse IP experiments, respectively.

### Cell proliferation assay

Antiproliferative effects of rMNh and rMCh, compared to that of rHco-gal-m, on PBMC were determined by performing cell counting kit-8 assay (Beyotime Biotechnology, Haimen, Jiangsu, China), as previously described [24]. Cells treated with the irrelevant purified empty recombinant pET-32a protein were used as negative controls. Cells in blank group were served as blank controls and the absorbance values at 450 nm (OD450) in blank controls were set as 100%. Cell proliferation index was calculated by the formula: OD450 group/OD450 blank control.

### Measurement of nitric oxide production

The release of NO was evaluated as previously described measuring intracellular nitrite in the PBMC by Griess reaction following the protocol of Total Nitric Oxide Assay Kit (Beyotime Biotechnology, Haimen, Jiangsu, China). Nitrite amount, proportional to the colored solution, was determined as absorbance at 540 nm (OD540) in each well using a microplate reader (Bio-Rad Laboratories, Hercules, California, USA). Absorbance values were converted to micromoles per liter using a standard curve that was generated by addition of 0–80 μmol/l sodium nitrite to fresh culture media. PBMC incubated with empty recombinant pET-32a protein were used as negative controls and PBMC without any treatment were set as a blank group.

### Apoptosis assay

Apoptosis in PBMC was evaluated and quantified by the flow cytometry (BD Biosciences, San Jose, California, USA) with the Annexin V-FITC kit (Miltenyi Biotec, Bergisch Gladbach, Nordrhein-Westfalen, Germany) as previously described [19]. Briefly, cells (1 × 10^6^ cells/ml) were treated with the presence of 40 μg/ml recombinant proteins (recombinant pET-32a protein, rMNh or rMCh) for 24 h and stained with Annexin V and propidium iodide (PI) according to the manufacturer’s instructions. PBMC without any treatment were set as blank controls.

### Transcriptional analysis

PBMC were activated with Concanavalin A (ConA, 10 μg/ml) and simultaneously cultured with recombinant pET-32a protein, rMNh and rMCh (40 μg/ml), at 37 °C for 24 h. PBMC only activated with ConA were set as a blank group. Total RNA was extracted and the resulting cDNA was synthesized according to the manufacturer’s specifications. The detection of cytokine transcription was conducted with standard procedure on the ABI 7500 Real-Time PCR System (Applied Biosystems, USA) with the specific primers for all targets and endogenous reference genes (Additional file 1: Table S3). The amplification efficiencies were verified to be similar (Additional file 1: Table S3) and the relative mRNA expression levels of target genes were calculated by the 2-ΔΔCt method. Each experiment was performed in triplicate.

### Statistical analysis

Statistical analysis for significant differences was performed using the Graphpad Premier 6.0 software package (Graphpad Prism, San Diego, California, USA) at *P* < 0.01. Data were expressed as the mean ± the standard deviation (SD).

## Results

### Binding of rMNh and rMCh to PBMC in vitro

**Fig. 1:**
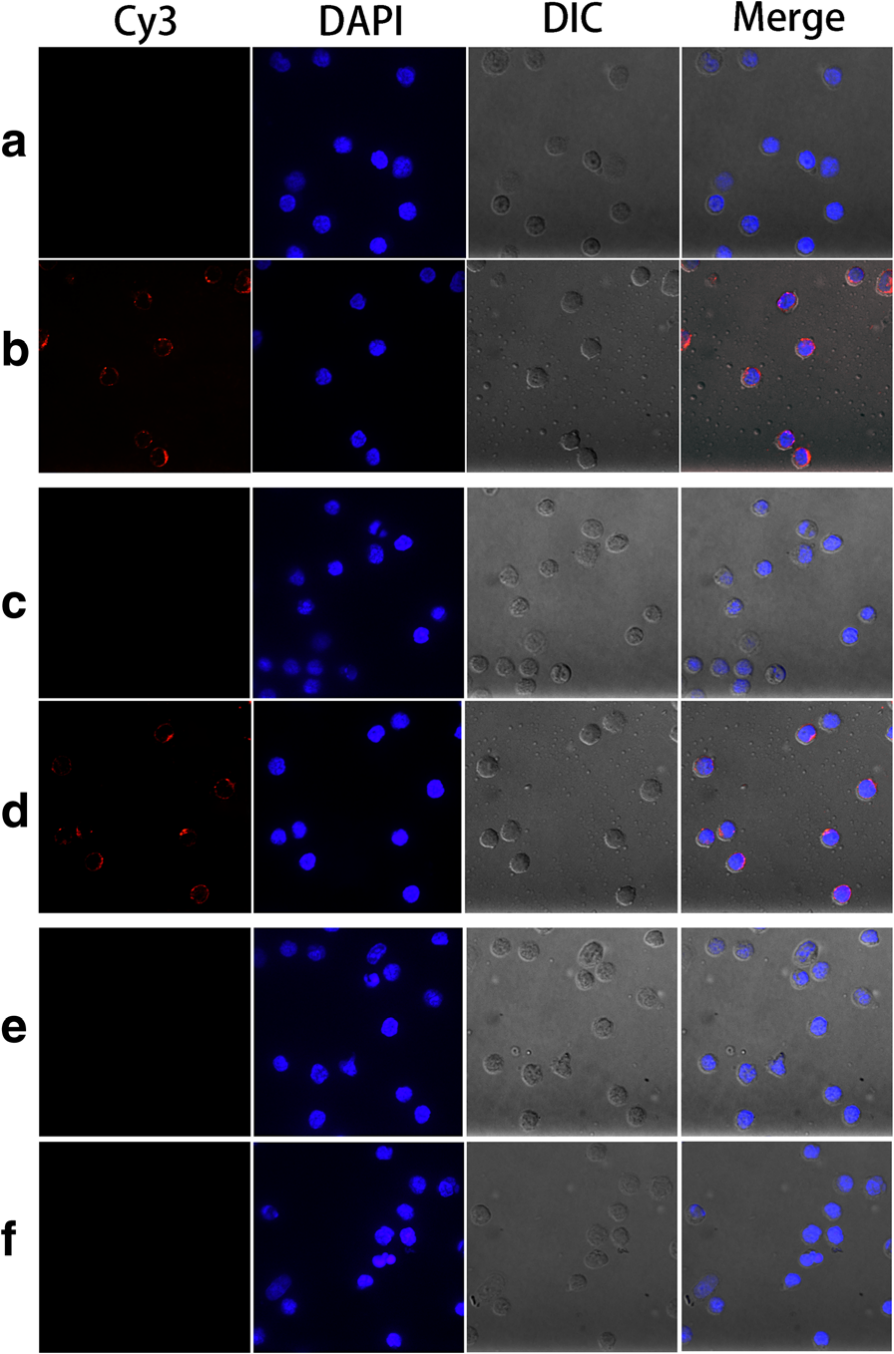
Binding of rMNh and rMCh to goat PBMC. The immunofluorescence assay was carried out by incubation of cells with rat anti-MNh/MCh IgG or negative rat IgG (Control). DAPI (blue) and Cy3-conjugated secondary antibodies (red) were utilized for double staining. Merge, overlap of Cy3, DAPI and DIC channels. **a** PBMC pretreated with rMNh were incubated with negative rat IgG (Control). **b** PBMC pretreated with rMNh were incubated with rat anti-MNh IgG. **c** PBMC pretreated with rMCh were incubated with negative rat IgG (Control). **d** PBMC pretreated with rMCh were incubated with rat anti-MCh IgG. **e** PBMC pretreated with empty recombinant pET-32a protein were incubated with negative rat IgG (Control). **f** PBMC pretreated with empty recombinant pET-32a protein were incubated with rat anti-pET-32a protein IgG

To investigate whether individual CRDs of Hco-gal-m may retain or exert their own biological activities, we checked the interaction of rMNh or rMCh with goat PBMC in vitro initially. Isolated PBMC were incubated with rMNh or rMCh and the binding was investigated by IFA. The Cy3-labeled rMNh or rMCh and the DAPI-labeled nuclei exhibited red and blue fluorescence, respectively. Intense red fluorescence was observed in the treated group (Fig. 1b, d) and no red fluorescence was detected in the control group (Fig. 1a, c, e, f), which indicated that both rMNh and rMCh could bind to the surfaces of PBMC.

### TMEM63A is a binding receptor of MNh, while TMEM147 is a binding receptor of MCh

**Fig. 2:**
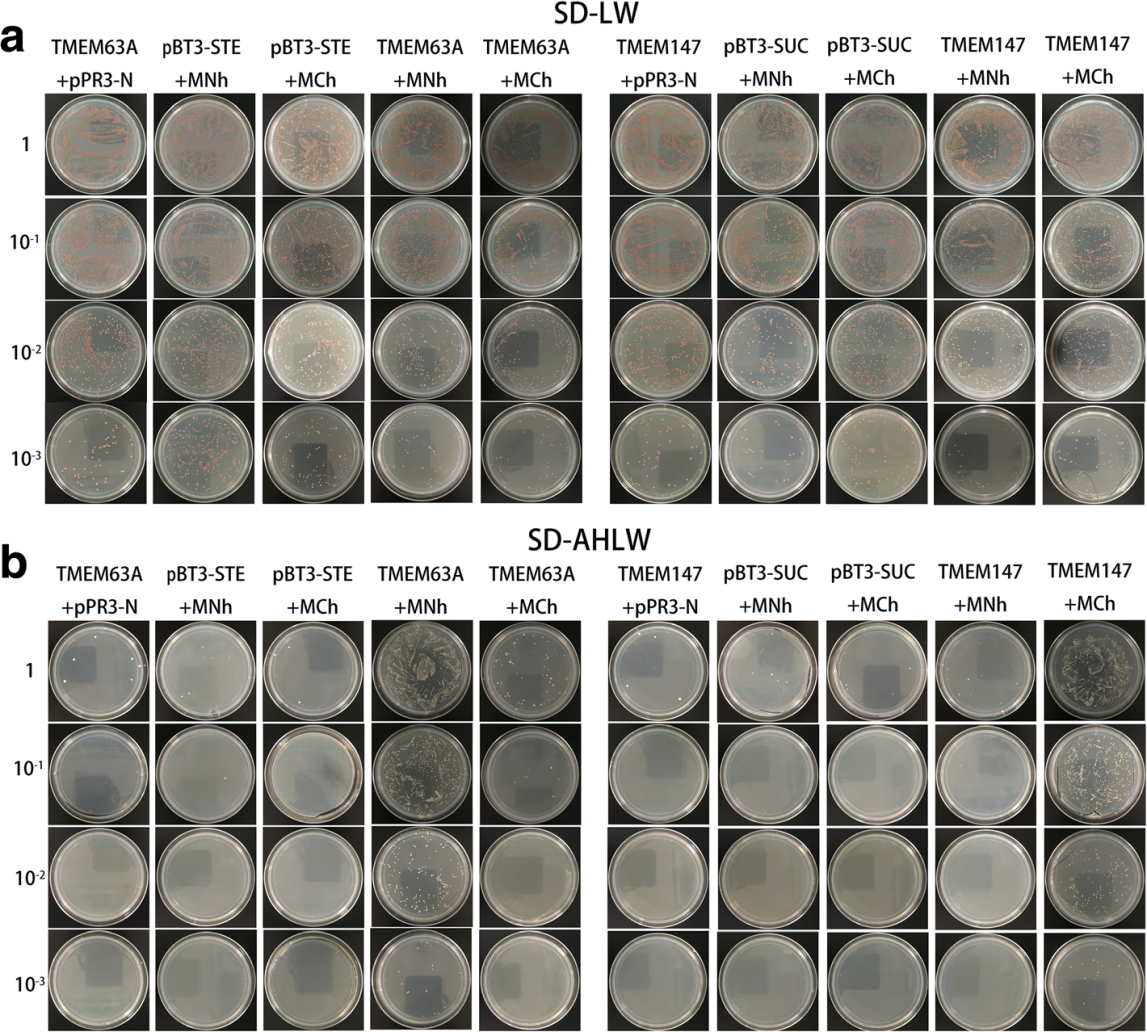
Testing protein-protein interaction of MNh to TMEM63A or TMEM147 and the interaction of MCh to TMEM63A or TMEM147 using DUAL membrane pairwise interaction assay. **a** Cells grown on control SD-LW block (without Leu and Trp) medium. **b** Cells grown on selective SD-AHLW block (without Ade, His, Leu and Trp). Yeast strain NMY51 carried each pairs of bait and prey plasmids (pBT3-STE, pBT3-SUC and pPR3-N are the control vectors with no cloned cDNA). The construct pairs of TMEM63A with pPR3-N, MNh with pBT3-STE, MCh with pBT3-STE, TMEM147 with pPR3-N, MNh with pBT3-SUC and MCh with pBT3-SUC were used as negative controls. The construct pairs of TMEM63A with MNh, TMEM63A with MCh, TMEM147 with MNh and TMEM147 with MCh were used as positive controls

Previous studies have demonstrated the interaction between Hco-gal-m to TMEM63A or TMEM147 [18, 19]. To detect the protein–protein interactions between MNh/MCh to TMEM147/TMEM63A, DUAL membrane pairwise interaction kit (Dualsystems Biotech, Schlieren, Switzerland), a variant of the YTH assay, was used in this study. If MNh/MCh fused to the C-terminal half of ubiquitin and TMEM147/TMEM63A fused to the N-terminal half of ubiquitin interacted, which resulted in reconstruction of the split ubiquitin, the reporter genes (HIS3 and ADE2) would allow the yeast strain to grow on SD-AHLW selective medium. Intriguingly, we found that when MNh was co-transformed with TMEM63A, or MCH was co-transformed with TMEM147, the yeast reporter strain NMY51 could grow on SD-AHLW (Fig. 2b). These observations showed that TMEM63A was a binding partner of MNh, while TMEM147 was a binding partner of MCh.

### Co-IP assays further indicated that MNh could bind to TMEM63A and MCh could bind to TMEM147

**Fig. 3:**
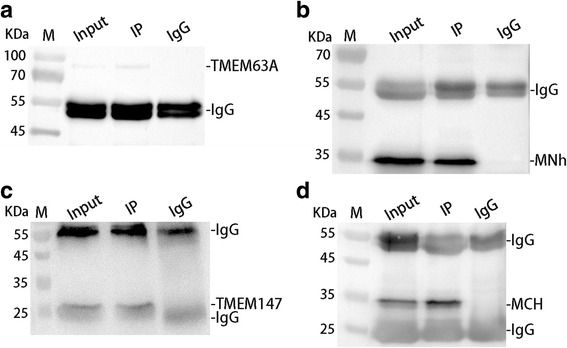
Co-IP assays further indicate that MNh can bind to TMEM63A and MCh can bind to TMEM147. Lane Input (**a**, **b**, **c**, **d**): Cell lysates were precipitated with rat anti-TMEM63A-NO IgG, rat anti-MNh IgG, rat anti-TMEM147-O IgG and rat anti-MCh IgG, respectively. Lane IP (**a**, **b**, **c**, **d**): Cell lysates were precipitated with rat anti-MNh IgG, rat anti-TMEM63A-NO IgG, rat anti-MCh IgG and rat anti-TMEM147-O IgG, respectively. Lane IgG (**a**, **b**, **c**, **d**): Cell lysates were precipitated with normal rat IgG. IP: immunoprecipitation. Immunoblot analysis using rat anti-MNh IgG and rat anti-MCh IgG demonstrated that rMNh can bind to TMEM63A and rMCh can bind to TMEM147. Lane M: marker

To further validate the results of YTH screening, independent co-IP assays were performed in rMNh or rMCh-stimulated goat PBMC. Consistent with the results of YTH assays, TMEM63A was detected in MNh immune complexes (IP) and in the PBMC lysates (Input), but not in rat normal IgG control group (Fig. 3a). Reciprocally, in the reverse co-IP assay, MNh was detected in TMEM63A immune complexes (IP) and in the PBMC lysates (Input), but not in the control group (Fig. 3b). So were TMEM147 in the forward co-IP assay (Fig. 3c) and MCh in the reverse co-IP assay (Fig. 3d). These observations suggest that the positive interactions of MNh with TMEM63A and MCh with TMEM147 in PBMCs were the results of specific binding.

### rMCh was much more potent than rMNh in inhibiting cell proliferation

**Fig. 4:**
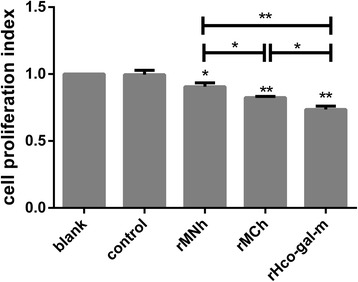
rMCh was much more potent than rMNh in inhibiting cell proliferation. PBMC were activated with ConA and incubated at the same time with 40 μg/ml recombinant proteins or recombinant empty protein pET32a (control) at 37 °C and 5% CO_2_. The proliferation was measured by CCK-8 incorporation after 72 h. Cell proliferation index was calculated considering the OD450 values in blank group as 100%. PBMC used for all replicates of distinct treatments in each experimental repetition were derived from the same goat. Results presented here are representative of three independent experiments. Data are presented as the mean ± SD, *P < 0.01, **P < 0.001 *vs* the control group, a capped line designates two groups that differ significantly (*P < 0.01, **P < 0.001)

The antiproliferative effects of rMNh and rMCh, compared to that of full-length rHco-gal-f, on PBMC in vitro were evaluated by performing cell counting kit (CCK8). No significant difference was observed between the blank group and the control group (ANOVA, *F*_(4, 10)_ = 74.04, *P* = 0.9993). The results showed that the proliferation of PBMC in the rMNh- (ANOVA, *F*_(4,10)_ = 74.04, *P* = 0.0050), rMCh- (ANOVA, *F*_(4,10)_ = 74.04, *P* < 0.0001) and rHco-gal-m-treated groups (ANOVA, *F*_(4,10)_ = 74.04, *P* < 0.0001) were significantly suppressed and inhibition by rHco-gal-m was more potent as compared to rMNh (ANOVA, *F*_(4,10)_ = 74.04, *P* < 0.0001) and rMCh (ANOVA, *F*_(4,10)_ = 74.04, *P* = 0.0053). Notably, the inhibition of PBMC proliferation in rMCh-treated group (ANOVA, *F*_(4,10)_ = 74.04, *P* = 0.0096) was much more significant than rMNh-treated group (Fig. 4).

### rMNh was much more effective than rMCh in suppressing nitric oxide production of PBMC

**Fig. 5:**
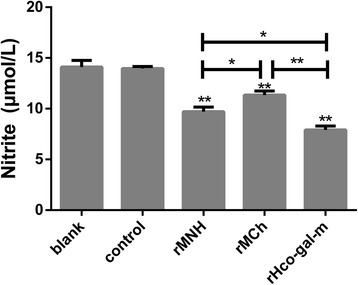
rMNh was much more effective than rMCh in suppressing nitric oxide production of PBMC. PBMC were activated with ConA and incubated at the same time with 40 μg/ml recombinant proteins or recombinant empty protein pET32a (control) at 37 °C and 5% CO_2_. The nitrite concentration was measured by using the Griess assay and used as an indicator of nitric oxide production by the PBMC. PBMC used for all replicates of distinct treatments in each experimental repetition were derived from the same goat. Results presented here are representative of three independent experiments. Data are presented as the mean ± SD, **P < 0.001 *vs* the control group, a capped line designates two groups that differ significantly (*P < 0.01, **P < 0.001)

Nitric oxide plays a crucial role in the host protection through either by limiting parasite growth or killing the parasites directly during parasitic infections [26]. Here, we investigated the effects of rMNh and rMCh on NO production of PBMC in comparison with rHco-gal-m by using the total nitric oxide assay kit. Results showed that no significant difference was observed between the blank group and the control group (ANOVA, *F*_(4,10)_ = 108.9, *P* = 0.9931). The release of NO in the rMNh- (ANOVA, *F*_(4,10)_ = 108.9, *P* < 0.0001), rMCh- (ANOVA, *F*_(4,10)_ = 108.9, *P* = 0.0002) and rHco-gal-m- (ANOVA, *F*_(4,10)_ = 108.9, *P* < 0.0001) treated groups were significantly reduced compared to the control group. Moreover, rHco-gal-m prevented NO production of PBMC with a higher efficacy than rMNh (ANOVA, *F*_(4,10)_ = 108.9, *P* = 0.0042) and rMCh (ANOVA, *F*_(4,10)_ = 108.9, *P* < 0.0001). In addition, rMNh (ANOVA, *F*_(4,10)_ = 108.9, *P* = 0.0082) had a stronger role in inhibiting NO production than rMCh (Fig. 5).

### rMCh was much more potent than rMNh in inducing PBMC apoptosis

**Fig. 6:**
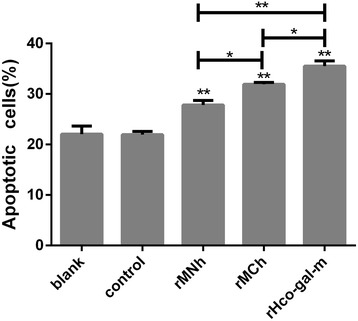
Apoptosis analysis of PBMC in response to rMNh, rMCh, and full-length Hco-gal-m by flow cytometry. Flow cytometric analysis of PBMC treated with recombinant proteins or recombinant empty protein pET-32a (control). Apoptosis of PBMC was determined by staining with annexin V and PI. The percentages of cells with different staining patterns are shown. The apoptosis rate was calculated by the percentage of early (AnnexinV + PI-) and late (AnnexinV + PI+) apoptotic PBMC. The percentage of apoptosis was measured on four separate occasions. PBMC used for all replicates of distinct treatments in each experimental repetition were derived from the same goat. Results presented here are representative of three independent experiments. Data are presented as the mean ± SD, **P < 0.001 *vs* the control group, a capped line designates two groups that differ significantly (*P < 0.01, **P < 0.001)

There have been many reports of galectin family members one common function of inducing apoptosis of various cell types [7, 27, 28]. To evaluate the effects of rMNh and rMCh on PBMC apoptosis, a cell apoptosis assay, using the externalization of phospholipid phosphatidylserine (PS) as a marker of cell apoptosis and positive DNA staining as an indicator for membrane leakage, was performed. The apoptosis rate was calculated by the percentage of early (AnnexinV + PI-) and late (AnnexinV + PI+) apoptotic PBMC. Flow cytometry analysis revealed that the treatments of rMHh (ANOVA, *F*_(4,10)_ = 138.0, *P* < 0.0001), rMCh (ANOVA, *F*_(4,10)_ = 138.0, *P* < 0.0001) and rHco-gal-m (ANOVA, *F*_(4,10)_ = 138.0, *P* < 0.0001) significantly increased the frequency of apoptotic PBMC compared to the control group and no significant change was observed between blank group and control group (ANOVA, *F*_(4,10)_ = 138.0, *P* = 0.9903). Meanwhile, there was a significant increase of apoptotic cells in the rHco-gal-m-treated group in comparison with the rMNh-treated group (ANOVA, *F*_(4,10)_ = 138.0, *P* < 0.0001) or rMCh-treated group (ANOVA, *F*_(4,10)_ = 138.0, *P* = 0.0010). Moreover, rMCh (ANOVA, *F*_(4,10)_ = 138.0, *P* = 0.0043) possessed a stronger apoptosis-inducing effect on PBMC than rMNh (Fig. 6).

### rMNh could inhibit the transcription of IFN-γ in vitro, but rMCh not

**Fig. 7:**
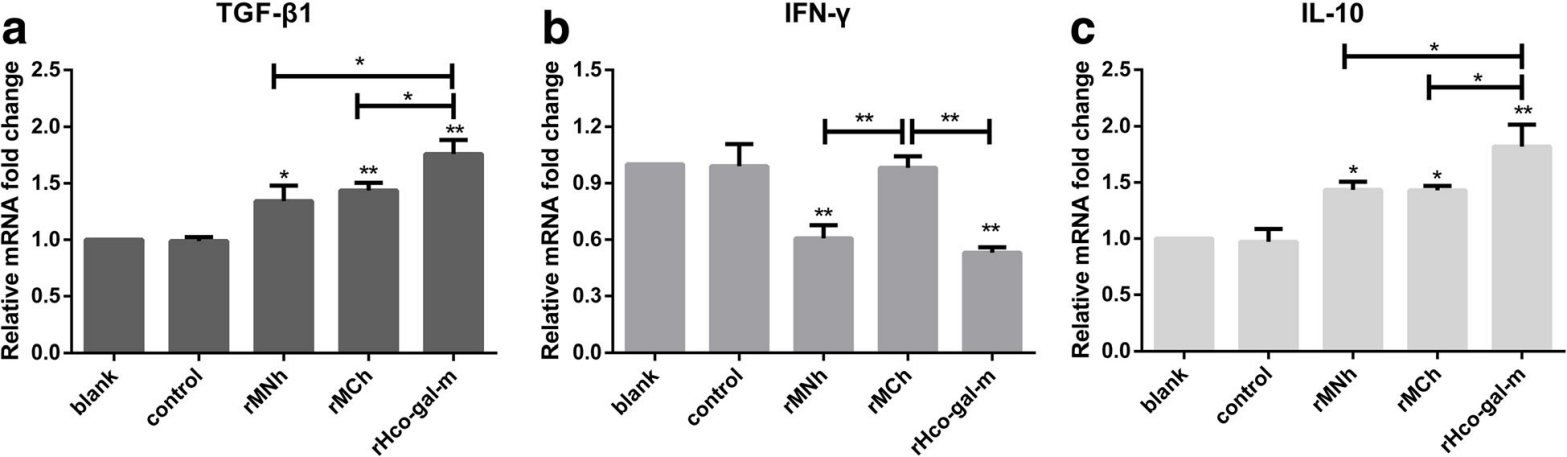
Relative levels of cytokine mRNA transcripts in goat PBMC treated with rMNh, rMCh, and full-length. PBMC were activated with ConA and simultaneously incubated in the absence of recombinant proteins or recombinant empty protein pET-32a (control), respectively. **a** TGF-β1. **b** IFN-γ. **c** IL-10. PBMC used for all replicates of distinct treatments in each experimental repetition were derived from the same goat. Results presented here are representative of three independent experiments. Data are presented as the mean ± SD, *P < 0.01, **P < 0.001 *vs* the control group, a capped line designates two groups that differ significantly (*P < 0.01, **P < 0.001)

Cytokines generated by the immune cells mediate the majority of the immune and inflammatory responses. Effects of rMNh and rMCh on the cytokine production in PBMC were evaluated by real-time PCR. The results indicated that co-incubation with rMNh (ANOVA, *F*_(4,10)_ = 31.70, *P* = 0.0028; *F*_(4,10)_ = 39.07, *P* = 0.0047), rMCh (ANOVA, *F*_(4,10)_ = 31.70, *P* = 0.0029; *F*_(4,10)_ = 39.07, *P* = 0.0008) and rHco-gal-m (ANOVA, *F*_(4,10)_ = 31.70, *P* < 0.0001; *F*_(4,10)_ = 39.07, *P* < 0.0001), respectively, dramatically increased the transcription of IL-10 and TGF-β1 in goat PBMC (Fig. 7a, c). Concurrently, rHco-gal-m was much more potent in the regulation of IL-10 and TGF-β1 transcription than either rMNh (ANOVA, *F*_(4,10)_ = 31.70, *P* = 0.0099; *F*_(4,10)_ = 39.07, *P* = 0.0015) or rMCh (ANOVA, *F*_(4,10)_ = 31.70, *P* = 0.0094; *F*_(4,10)_ = 39.07, *P* = 0.0088), whilst no significant influence was observed between rMNh-treated group (ANOVA, *F*_(4,10)_ = 31.70, *P* > 0.9999; *F*_(4,10)_ = 39.07, *P* = 0.7163) and rMCh-treated group (Fig. 7a, c). In addition, we noted that the transcription of IFN-γ was prominently suppressed by rMNh (ANOVA, *F*_(4,10)_ = 35.19, *P* = 0.0003) and rHco-gal-m (ANOVA, *F*_(4,10)_ = 35.19, *P* < 0.0001), compared to the control group, while no significant change was observed between rMNh-treated group (ANOVA, *F*_(4,10)_ = 35.19, *P* = 0.6646) and rHco-gal-m -treated group (Fig. 7b). Interestingly, the transcription of IFN-γ was not affected by rMCh (ANOVA, *F*_(4,10)_ = 35.19, *P* = 0.9998) in comparison with the control group (Fig. 7b).

## Discussion

With two homologous but distinct domains, the tandem-repeat galectins can oligomerize by association of the C-terminal CRD of one galectin with the C-terminal CRD of another galectin and association of the N-terminal CRD with another N-terminal CRD. This oligomerization form of large multimers containing numerous CRDs enables TR galectins to bind multifarious ligands [3, 29, 30]. Furthermore, given this association, individual N- and C-terminal CRD of tandem-repeat galectins have the potential to form oligomers, a fashion analogous to the homodimerization of prototype galectins, to exert their own biological activity [21, 31–33]. In this study, either rMNh or rMCh bound strongly to goat PBMC and these bindings, which were confirmed subsequently by protein-protein interaction analysis, may trigger transmembrane signaling transduction to modulate host immune response.

Although galectins are characterized by affinity for β-galactosides containing glycans, they have substantial diversity in recognition properties. More specifically, endogenous ligands including CD7, CD44, CD45, CD71, GM1, MUC1, TIM3, CTLA4, MUC16 and MerTK have been identified by galectin family [34–39]. However, there is very limited information regarding the host ligands of parasitic galectins. As exogenous galectins, the parasitic galectins may form multivalent complexes with host ligands to deliver CRD-dependent intracellular signals that modulate downstream effector functions [3, 8, 40]. In this study, YTH screening and co-IP assays demonstrated that TMEM63A was a binding receptor of MNh, while TMEM147 was a binding receptor of MCh. Both TMEM147 and TMEM63A are novel, ubiquitously expressed polytopic membrane proteins with multiple transmembrane structures. They are highly conserved among mammals and their physiological roles are still being elucidated. Dettmer et al. [41] demonstrated that TMEM147 was a genuine component of the Nicalin-NOMO (Nicastrin-like protein-Nodal modulator) protein complex which could alter Nodal signaling. Moreover, Nodal is a member of the transforming growth factor beta superfamily, which regulates cell proliferation and controls cell-fate specification and differentiation [42]. Rosemond et al. [43] suggested that TMEM147, as a binding partner of the M3 muscarinic acetylcholine receptor (M3R), was a potent negative regulator of M3R mediated stimulatory effects of carbachol on H508 cell proliferation and p90RSK activation. Combined, these data indicate that TMEM147 may involve in the regulation of some cellular function including cell proliferation. TMEM63 proteins were the mammalian orthologues of AtCSC1 and OSCA1, two integral membrane proteins both which were essential for hyperosmolality induced Ca^2+^ signaling in *Arabidopsis* [44]. Zhao et al. [45] demonstrated that all the members of TMEM63 proteins, TMEM63A, TMEM63B and TMEM63C, constituted a hyperosmolarity activated ion channel. Furthermore, Yadav et al. [46] demonstrated that the CRD of Gal/GalNAc Lectin expressed by *Entamoeba histolytica* bind to Calcium to modulate host cell adhesion. Considered together, TMEM63A could be the calcium ion channel for MNh to exert its own function.

Given that the individual MNh and MCh display conserved amino acid with 32% similarity to each other, they may exhibit similar or different functions in the immune evasion of *H. contortus* mediated via Hco-gal-m. To further elucidate the specificities of each CRD, we detect the influence of rMNh, rMCh and the full-length Hco-gal-m on PBMC independently. In this study, we revealed that MNh had the dominant effect to inhibit the transcription of IFN-γ and was more effective in inhibiting NO production of PBMC, while MCh was much potent in suppressing cell proliferation and inducing apoptosis. As mentioned before, galectins exhibit the dizzying array of opposing functions characterizes; host galectins can prevent pathogen binding to host target cells and have direct microbicidal activity on parasites, while parasitic galectins can enhance pathogen production and promote immune evasion. The effect was determined by which host and parasite ligands are bound by a specific galectin and then it determines the downstream consequences [3]. Interestingly, we find that the two CRDs of Hco-gal-m contribute differently to its immunomodulatory functions in host-parasite interaction. All of the outcomes may be due to the binding of MNh to TMEM63A and MCh to TMEM147 which caused similar, but not completely equivalent, downstream effects contributing to parasitic immune evasion. Nitric oxide is produced by macrophages activated mainly by the secretion of pro-inflammatory cytokines including IFN-γ [26]. Inhibition of IFN-γ transcription, caused by the binding of MNh with TMEM63A, may significantly downregulate NO production of PBMC, which could be one of the reasons why rMNh play a stronger role in inhibiting NO production. In most cases, TGF-β/Smad signaling pathway could restrain cancer cell growth by downregulation of proliferation, prevention of metastasis and induction of apoptosis [47]. Thus, the modulation of Nodal signaling related to TGF-β/Smad signaling pathway resulting from the bindings of MCh with TMEM147 could also inhibit cell proliferation and induce host immune cell apoptosis dramatically. This could be one of the mechanisms of Hco-gal-m to facilitate the immune evasion.

In our previous studies, Yuan et al. [18] and Yan et al. [19] found that the interaction of Hco-gal-m/f with TMEM63A or TMEM147 played similar roles in inhibiting cell proliferation, phagocytosis, nitric oxide production and enhancing the transcription of TGF-β1 and IL-10, but different roles in promoting apoptosis and suppressing cell migration. This could also due to the binding of MNh to TMEM63A and MCh to TMEM147. Consistent with this rule which determined the effect of galectins on cells, it is not hard to understand why the interaction of Hco-gal-m with TMEM63A play a stronger role in the regulation of cell migration, whilst the interaction of Hco-gal-m with TMEM147 play a greater role in cell apoptosis. However, the detailed functions of TMEM63A or TMEM147 and their downstream binding molecules, along with associated signaling pathways, need to be further investigated.

The N-terminal and C-terminal CRDs of tandem-repeat galectins are connected by a single polypeptide chain, called the linker domain [48]. Recent studies with tandem-repeat galectins have speculated the role of linker region, including protein-protein interactions, membrane insertions and regulation of CRD presentations [49–51]. Furthermore, the linker domain may mediate the intermolecular interaction of the CRDs, resulting in inducing a specific biological response at a greater potency [52]. Thus, the existence of the linker domain may be indispensable. In this study, we found that full-length rHco-gal-m gave higher capabilities to modulate cytokine secretions, promote PBMC apoptosis, inhibit cell proliferation and NO production than any single CRDs. Taken together, these suggest that the completely biological functions of Hco-gal-m require a complete structure, both the two CRDs and linker region.

## Conclusion

In this study, we examined the biological basis of individual domains of Hco-gal-m for the first time. A comparison of the ability of MNh and MCh to suppress PBMC proliferation, induce apoptosis, inhibit NO production, and alter cytokine transcription showed that MNh and MCh contribute differently to the multiple functions of Hco-gal-m. The different binding specificities, MNh withTMEM63A or MCh with TMEM147, may partially explain their different roles in immune regulation. These results will provide new insights into the mechanisms of Hco-gal-m involved in immune evasion by nematodes. However, the underlying mechanisms of structure-function relationship of Hco-gal-m need further investigation.

## Additional files

Additional file 1: Table S1. Primer sequences for PCR amplification. **Table S2.** Primer sequences for yeast two-hybrid screening. **Table S3.** Primer sequences for real-time PCR. (DOCX 15 kb)

Additional file 2: Figure S1. The purification of rMNh and rMCh. Protein samples were resolved by SDS–PAGE on 12% of polyacrylamide gel and stained with coomassie brilliant blue R250. M: standard protein molecular marker; Lane 1: soluble extract of cultured cells for rMNh; Lane 2: purified recombinant MNh was approximately 34.5 kDa; Lane 3: soluble extract of cultured cells for rMCh; Lane 4: purified recombinant MCh was approximately 34.0 kDa. (DOCX 809 kb)

## Abbreviations

BG B^+^*E. coli*: blood group B positive *Escherichia coli*
co-IP: Co-immunoprecipitation
ConA: Concanavalin A
CRD: Carbohydrate recognition domain
DAPI: 2-(4-amidinophenyl)-6-indole carbamidinedihydrochloride
GBP: Glycan-binding protein
IFA: Immunofluorescence assay
IFN-γ: Interferon-γ
IL-10: Interleukin-10
IPTG: Isopropyl-β-D-thiogalactopyranoside
LPS: Lipopolysaccharide
M3R: M3 muscarinic acetylcholine receptor
MCh: C-terminal CRD of Hco-gal-m
MNh: N-terminal CRD of Hco-gal-m
Nicalin-NOMO: Nicastrin-like protein-Nodal modulator
NO: Nitric oxide
PBMC: Peripheral blood mononuclear cells
PI: Propidium iodide
PS: Phospholipid phosphatidylserine
RNAi: RNA interference
SD: Sprague Dawley
TGF-β1: Transforming growth factor-β1
TMEM147: Transmembrane protein 147
TMEM63A: Transmembrane protein 63A
TR: Tandem-repeat
YTH: Yeast two-hybrid
