# Supplementary material for: The N- and C-terminal carbohydrate recognition domains of Haemonchus contortus galectin bind to distinct receptors of goat PBMC and contribute differently to its immunomodulatory functions in host-parasite interactions
Source: Parasit Vectors. 2017 Sep 5;10:409. doi: 10.1186/s13071-017-2353-8 (PMC5584048; doi:10.1186/s13071-017-2353-8)
Supplement: Supplementary file 1 — Primer sequences for PCR amplification. Table S2. Primer sequences for yeast two-hybrid screening. Table S3. Primer sequences for real-time PCR. (DOCX 15 kb) [file 13071_2017_2353_MOESM1_ESM.docx]

**Table S1. Primer sequences for PCR amplification**

| Gene Name | Primer Sequence (5’-3’) |
| --- | --- |
| MNh | CCGgaattcATGGTACCCTACCGCAGCTTG (*Eco*R Ⅰ) |
|  | CCCaagcttTCATCCCCAGTGGACGTGG(*Hind* Ⅲ) |
| MCh | CCGgaattcATGCCATACGAGAGTGGAATCG (*Eco*R Ⅰ) |
|  | CCCaagcttCTACTGGATCTGGATGCCAGTCA (*Hind* Ⅲ) |

**Table S2. Primer sequences for yeast two-hybrid screening**

| Gene Name | Primer Sequence (5’-3’) |
| --- | --- |
| MNh | CCTGCATggccattacggccATGGTGTCACAGTTCCTACACTGG (*Sfi* I) |
|  | TATCGACggccgaggcggccgGACTGGATAGTATTTTCCTCCCCA (*Sfi* I) |
| MCh | CCTGCATggccattacggccATGGGAAAATACTATCCAGTCCC (*Sfi* I) |
|  | TATCGACggccgaggcggccgTGGATCTGGATGCCAGTCAG (*Sfi* I) |
| TMEM63A | CCTGCATggccattacggccATGACCGACTCCCCTTTCCTG (*Sfi* I) |
|  | TATCGACggccgaggcggccAAGTCCTCCTGGTCGGCGG (*Sfi* I) |
| TMEM147 | CCTGCATggccattacggccATGACGCTTTTCCACTTCGGG (*Sfi* I) |
|  | TATCGACggccgaggcggccAAGGAGTGCACGTTGACAACGG (*Sfi* I) |

**Table S3. Primer sequences for real-time PCR**

| Gene Name | | Primer Sequence (5’-3’) | Amplification efficiency (%)* | Correlation coefficients (r^2^) |
| --- | --- | --- | --- | --- |
| beta-actin | | CACCACACCTTCTACAAC | 95.41 | 0.9991 |
|  | TCTGGGTCATCTTCTCAC | |  |  |
| IL-10 | CCTTGTCGGAAATGATCCAG | | 98.68 | 0.9993 |
|  | AGGGCAGAAAACGATGACAG | |  |  |
| IFN-γ | GAACGGCAGCTCTGAGAAAC | | 98.02 | 0.9982 |
|  | GGTTAGATTTTGGCGACAGG | |  |  |
| TGF-β1 | CATGAACCGGCCCTTCCT | | 98.98 | 0.9996 |
|  | GAAGTCAATGTAGAGCTGACGAACA | |  |  |

^*^ Amplification efficiency (%) = (10^-1/slope^ -1) ×100
