# Supplementary material for: The N- and C-terminal carbohydrate recognition domains of Haemonchus contortus galectin bind to distinct receptors of goat PBMC and contribute differently to its immunomodulatory functions in host-parasite interactions
Source: Parasit Vectors. 2017 Sep 5;10:409. doi: 10.1186/s13071-017-2353-8 (PMC5584048; doi:10.1186/s13071-017-2353-8)
Supplement: Supplementary file 2 — The purification of rMNh and rMCh. Protein samples were resolved by SDS–PAGE on 12% of polyacrylamide gel and stained with coomassie brilliant blue R250. M: standard protein molecular marker; Lane 1: soluble extract of cultured cells for rMNh; Lane 2: purified recombinant MNh was approximately 34.5 kDa; Lane 3: soluble extract of cultured cells for rMCh; Lane 4: purified recombinant MCh was approximately 34.0 kDa. (DOCX 809 kb) [file 13071_2017_2353_MOESM2_ESM.docx]

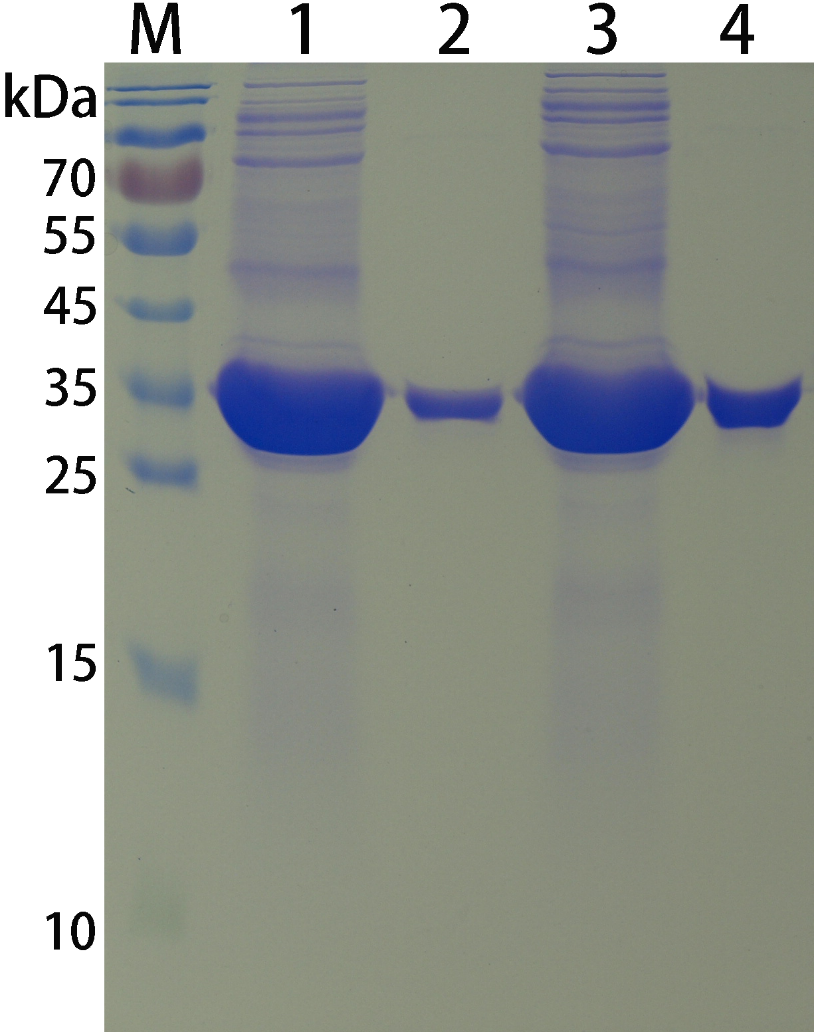


**Figure S1. The purification of rMNh and rMCh.** The purification of rMNh and rMCh. Protein samples were resolved by SDS–PAGE on 12% of polyacrylamide gel and stained with coomassie brilliant blue R250. M: standard protein molecular marker; lane 1: soluble extract of cultured cells for rMNh; lane 2: Purified recombinant MNh was approximately 34.5 kDa; lane 3: soluble extract of cultured cells for rMCh; lanes 4: Purified recombinant MCh was approximately 34.0 kDa;
